# Nanostructure Introduces Artifacts in Quantitative Immunofluorescence by Influencing Fluorophore Intensity

**DOI:** 10.1038/s41598-017-00447-7

**Published:** 2017-03-27

**Authors:** Christopher A. R. Chapman, Xiangchao Zhu, Hao Chen, Ahmet A. Yanik, Pamela J. Lein, Erkin Seker

**Affiliations:** 1 Department of Biomedical Engineering, University of California – Davis, Davis, CA 95616 USA; 2 Department of Electrical Engineering, University of California – Santa Cruz, Santa Cruz, CA 95064 USA; 3 Department of Molecular Biosciences, University of California – Davis, Davis, CA 95616 USA; 4 Department of Electrical & Computer Engineering, University of California – Davis, Davis, CA 95616 USA

## Abstract

Quantitative analysis of fluorescence signals from cells reacted with fluorescently labeled probes is a widely-used method for assessing cell biology. This method has become especially powerful for screening novel nanostructured materials for their influence on cell behavior. However, the effect of nanostructured surface on fluorescence intensity has largely been ignored, which likely leads to erroneous conclusions about cell behavior. This paper investigates this possibility by using fibroblasts cultured on nanoporous gold (np-Au) as a model nanostructured material system. We found that fibroblasts stained for f-actin using phalloidin conjugated with common fluorophores display different levels of fluorescence on np-Au, planar gold, and glass, suggesting different levels of f-actin composition. However, direct quantification via western blots indicates that the actin expression is the same across all conditions. We further investigated whether the fluorescence intensity depended on np-Au feature size, complementing the findings with reflection dark field measurements from different np-Au surfaces. Overall, our experimental measurements in agreement with our electrodynamic simulations suggest that nanostructured surfaces alter the fluorescence intensity of fluorophores by modulating both the excitation and light emission processes. We conclude that comparison of fluorescence on materials with different nanostructures should be done with a quantification method decoupled from the nanostructure's influence.

## Introduction

Quantitative immunofluorescence is an essential technique for investigating cell biology (e.g., inflammatory state, metabolic activity, expression of specific cellular products) in a wide range of applications, including evaluation of the biocompatibility of materials, drug screening and fundamental biological studies^1–3^. With the advent of nanotechnology, nanostructured materials have attracted significant use as biomaterials due to many desirable features, such as high surface area-to-volume ratio for drug loading, topographical cues for guiding neuronal processes, and both soluble and topographical cues for facilitating differentiation of stem cells^4–8^. In order to evaluate the biological properties of these materials, quantitative immunofluorescence is commonly used to determine levels of cellular proteins via fluorescent probes specific to a target molecule, which can provide information on cellular responses to underlying substrates (e.g., reactivity, apoptosis, cellular differentiation, etc.). However, fluorescence intensity variations due to the far-field optical effects on nanostructured surfaces are routinely neglected. For example, assume that cells grown on two different nanostructured surfaces express the same level of a protein. If one uses a direct method such as western blotting to quantify protein levels, the conclusion would be that there is no statistical difference between the cellular response to the two surfaces. On the other hand, fluorescence microscopy measurements on different substrates may yield different results due to purely optical effects, that is, not because the protein levels are different, but because the nanostructured surfaces influence the observed fluorescence signal. Thus, an erroneous conclusion would be reached using fluorescence readouts. Nanostructuring of material surfaces are known to significantly alter light interactions at the surface. In fact, previous studies have reported both quenching and enhancement of fluorescence signal due to the near-field effects^9, 10^. However, far-field phenomenon, such as spectrally changing light reflectance and scattering due to localized surface plasmon resonances (LSPR)^11^ and bulk plasmonic effects on metal surfaces can also significantly change fluorescence signal. In the case of cell-conjugated fluorophores, the fluorescence signal variations are largely far-field effects since the amount of fluorophores located within the hot spots on/in nanoporous surface constitute a negligibly small fraction of the total fluorophores. Therefore, near-field enhanced signals have minor contributions to overall fluorescence signal. The goal of this study is to determine whether nanostructured surfaces introduce artifacts in cell-conjugated fluorophores through far-field effects. Here, we systematically study the protein expression in fibroblasts cultured on different nanostructure materials using a combination of quantitative immunofluorescence, western blotting, and reflection dark field spectroscopy. Our experimental findings show strong agreement with our rigorous 3-D finite difference time domain (FDTD) simulations based on first principles solution of Maxwell’s equations.

## Results

**Figure 1:**
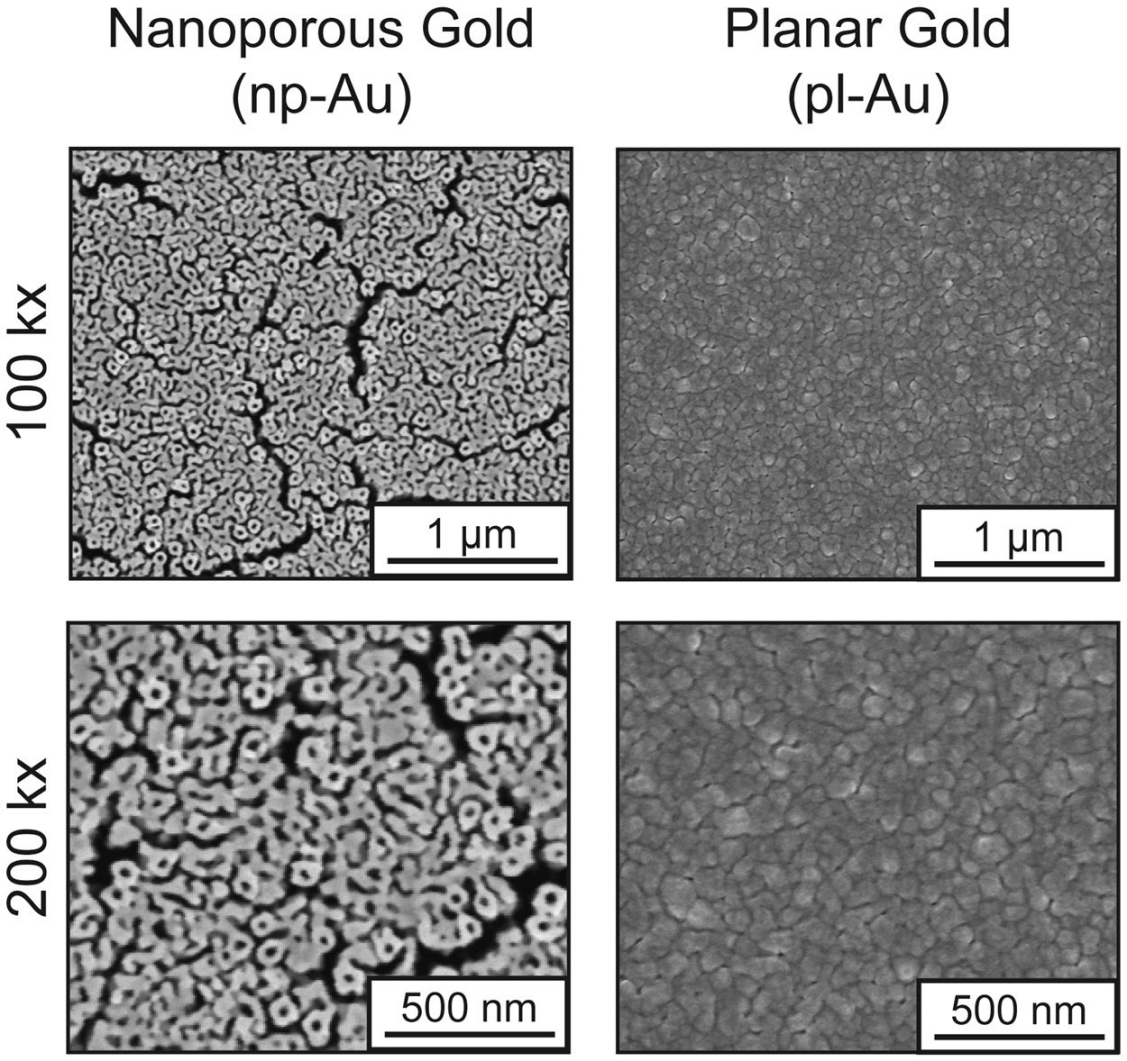
Low (top) and high (bottom) magnification scanning electron micrographs of the standard nanoporous gold (left) and planar gold (right) morphologies used in the following studies.

In order to establish a preliminary comparison between directly-measured protein content (western blot) and indirectly-measured protein content (fluorescence intensity), NIH/3T3 murine fibroblasts were grown to day *in vitro* 7 on nanoporous gold (np-Au), unstructured planar gold (pl-Au), and glass substrates. Nanoporous gold is a versatile model nanostructured material as its feature size can be tuned by several techniques^12, 13^ and gold is a common material system for biomaterial applications^14^. The thin film np-Au used in this study consists of a bi-continuous network of pores and gold ligaments with feature sizes in the range of 87 (average diameter) and 30 (average width) nanometers respectively (Fig. 1)^15, 16^. Additionally, due to the significant knowledge on light interactions with nanostructured gold systems^9, 17^ (and np-Au specifically^18–22^) this material constitutes easier to characterize model system for these experiments.

**Figure 2:**
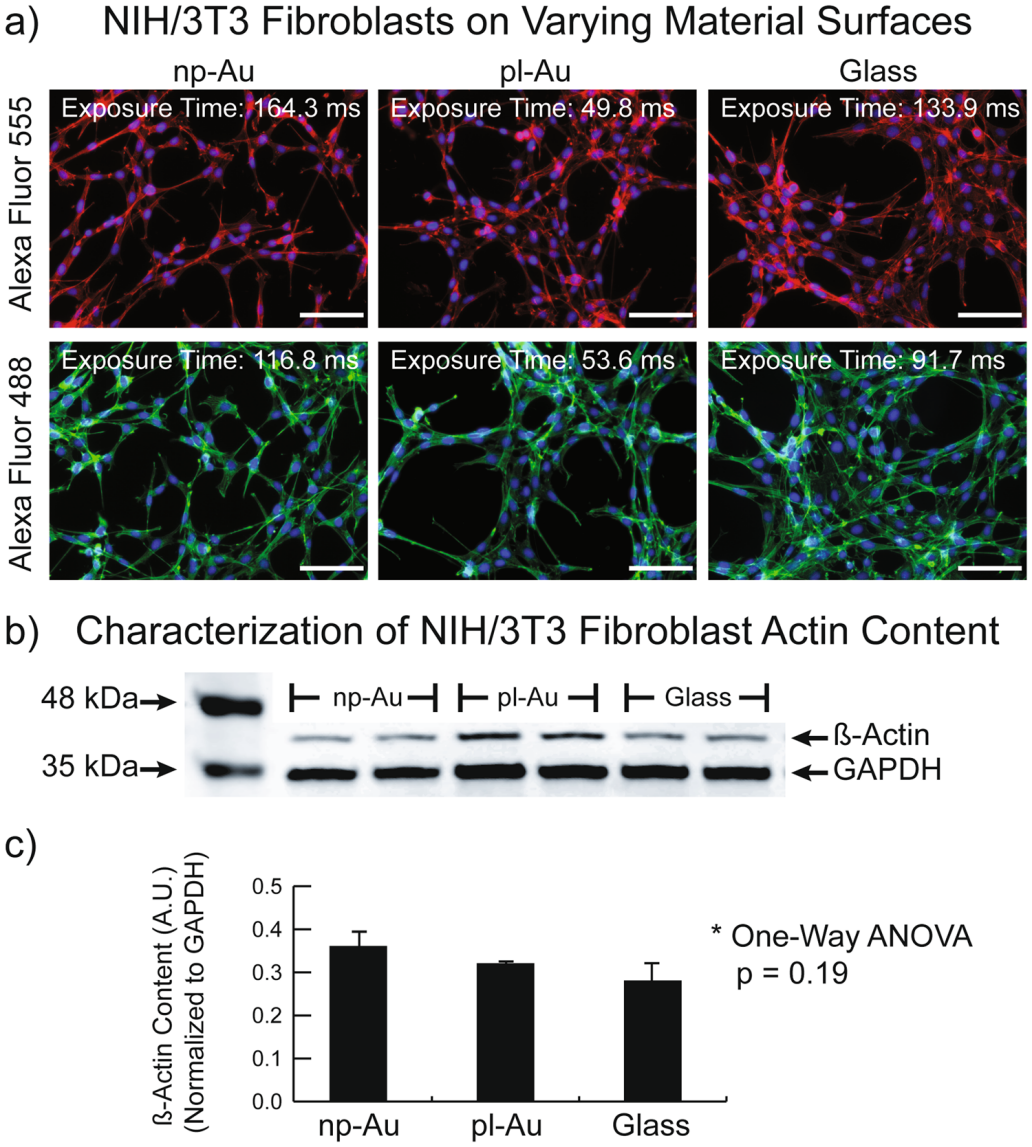
(**a**) Fluorescence micrographs of actin stained fibroblasts on varying surfaces illustrate similar cell coverage on each material but drastically different exposure times to normalize the image intensity (scale bar corresponds to 50 µm). (**b**) Representative western blot of the actin and GAPDH content in fibroblasts grown on the three different materials. (**c**) Quantification of the mean beta-actin intensity normalized to mean GAPDH intensity (between the two lanes pictured in Fig. 2b of the western blot data) demonstrates that there is no significant difference in protein expression between fibroblasts grown on each material (error bars represent standard deviation). Full-length blots/gels are presented in Supplementary Figure S2.

The f-actin content in fibroblasts cultured on these different surfaces was quantified by fluorescence intensity utilizing fluorophore-tagged phalloidin (Alexa Fluor 488 and 555) (Fig. 2a). In parallel cultures, the actin content was determined by western blotting using an antibody specific for beta-actin (normalized to glyceraldehyde 3-phosphate dehydrogenase (GAPDH) content) (Fig. 2b). Through this cross-comparison of protein levels, we effectively decoupled the measured results from cell type. Statistical comparison of the western blot analysis of beta-actin content normalized to GAPDH did not display any significant difference between the three different samples (ANOVA p = 0.19) (Fig. 2c).

**Figure 3:**
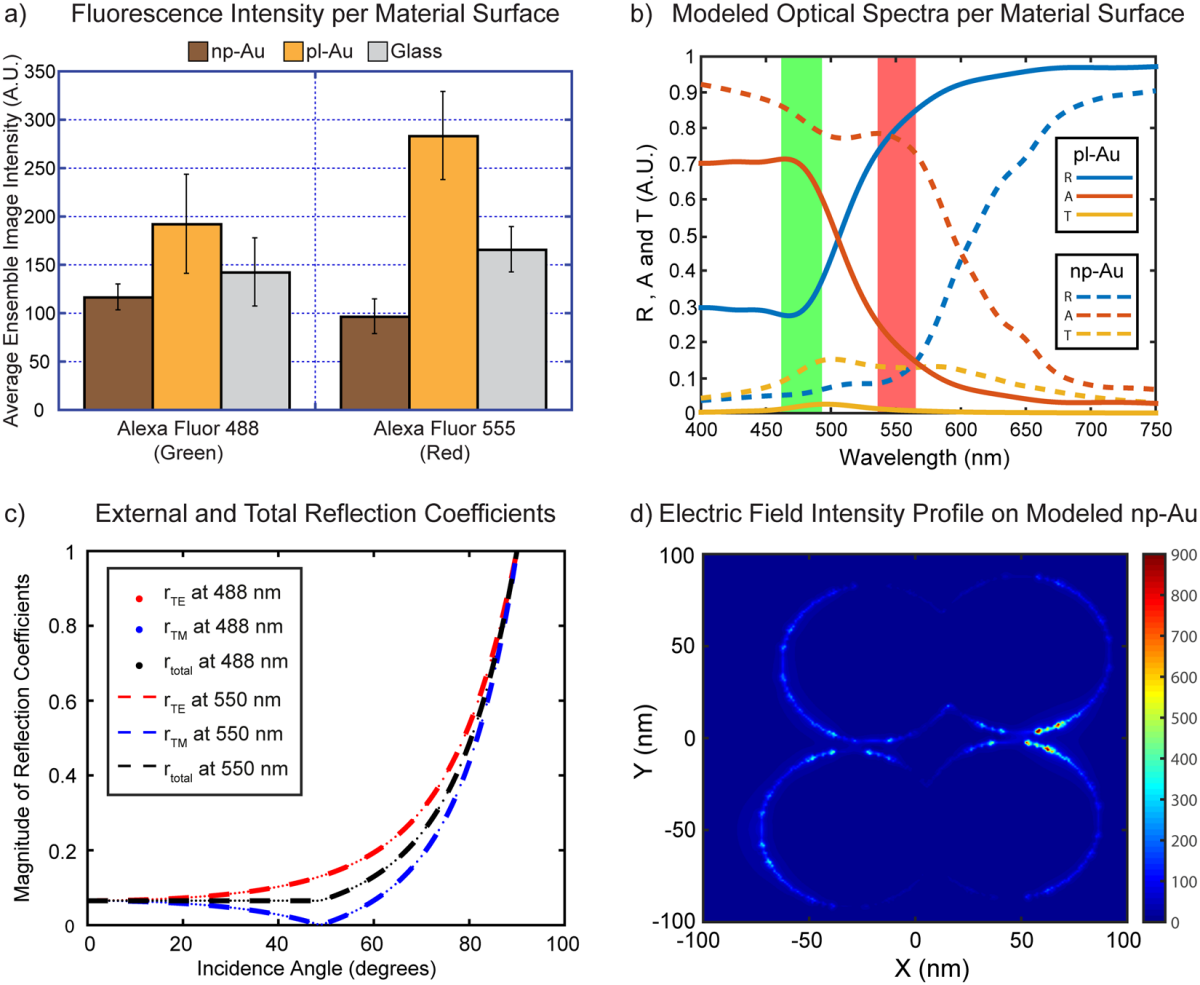
(**a**) The mean ensemble-average fluorescence intensity (error bars represent standard deviation) from images taken at a constant exposure time (100 ms) from three independent samples (for each condition) shows both an overall decrease in intensity between green and red fluorophores on the np-Au and pl-Au, as well as a decrease in intensity between np-Au and pl-Au. (**b**) Absorption, reflection, and transmission spectra for pl-Au and np-Au show that the difference in overall intensity observed between the two fluorophores is due to the changing absorption and reflection spectra for gold in the tested wavelengths. (**c**) External reflection coefficients for different polarization states of light r_TE_ and r_TM_, together with total reflection coefficient r_total_ (r_total _ = (r_TE_ + r_TM_)/2) are shown for varying angle of incidence at two different wavelengths 488 nm and 550 nm. Here, Fresnel's relations are used for the water/glass interface. (**d**) Strongly enhanced near-field intensity profile at 550 nm on the top gold surface reveals the near-field enhancement effects around the rims of the nanopores due to the excitation of LSPRs.

In order to calculate the average fluorescence intensity of f-actin stained fibroblasts on each different material surface, a custom written MATLAB script was used to calculate an ensemble-averaged intensity value from constant (100 millisecond) exposure images while normalizing it to the cellular surface coverage (see code in Supporting Information). Interestingly, in contradiction with the results obtained using western blotting, the fluorescence intensity of actin-stained fibroblasts grown on np-Au, pl-Au, and glass was significantly different and varied according to the nature of the material surface and the excitation/emission wavelength of the fluorophore (Fig. 3a). Most notably, the cells on np-Au exhibited a 3-fold attenuation in fluorescence intensity compared to those on pl-Au for the red fluorophore (Alexa Fluor 555). The intensity of the green fluorophore (Alexa Fluor 488) also decreased slightly (~1.5-fold) on np-Au in comparison to both pl-Au and glass. The transmission, reflection, and absorption spectra were calculated for glass, pl-Au and np-Au samples by solving 3-D Maxwell equations using finite-difference time-domain (FDTD) simulations^23^. Optical response (i.e., absorption, transmission and reflection) of the glass and gold surfaces are shown within a broad wavelength range spanning from 400 nm to 750 nm in Fig. 3b. Dielectric parameters for pl-Au are obtained from previous experimental measurements^24^. Excitation bands (determined by the filter cubes used in the experiments) are indicated with green and red columns for Alexa Fluor 488 and Alexa Fluor 555, respectively. Stronger absorption (red curve) and diminished reflection (blue curve) was observed for incident light for wavelengths shorter than 550 nm for the pl-Au surface, while a nearly wavelength independent reflectance and transmission is observed for glass surfaces. Spectral behavior of reflectance and absorption on pl-Au surfaces are attributed to increased absorption of high energy photons (in excess of ~1 eV) due to the inter-band excitation of electrons in gold substrate^24^. In the green spectrum (450–490 nm) corresponding to excitation of Alexa Fluor 488, the absorption of gold film is 50% higher than the reflection on average; whereas in the red spectrum (538–562 nm) corresponding to excitation of Alexa Fluor 555, the reflection of the Au film is 400% higher than the absorption on average (Fig. 3b). Therefore, stronger fluorescence signals observed for Alexa Fluor 555 labeled cells on Au surfaces are attributed to the stronger reflectance of gold in longer wavelengths (538–562 nm) in agreement with experimental measurements. Similarly, far-field optical characteristics of the np-Au surfaces are calculated using FDTD simulations as shown in Fig. 3c. Nanoporous material characteristics are incorporated into the simulations by adding randomly distributed water spheres of 87 nm in diameter in a pristine gold layer with a thickness 100 nm. A diameter of 87 nm for the spheres was chosen to accurately depict the average pore size of the np-Au films used in this study. A random generator script is used to mimic the 3D percolation, while the filling ratio is controlled using a volume ratio of V_percolation_/V_Au_ through changing the number of waterholes per unit volume. For a filling factor of about 51% a strong drop in reflectance signal is observed at both fluorescence excitation wavelengths in agreement with experimental measurements. Enhanced absorption and diminished reflectance is a result of both inter-band excitation of electrons in gold material and the excitation of LSPRs within the np-Au layer. As shown in Fig. 3d, enhanced near-fields created around the nanopores within the Au film is a result of excitation of LSPRs. Similar to gold nanoparticles as reported in previous studies^25^, LSPRs within a spectral window spanning from 450–550 nm (depending on feature sizes) are observed^18, 26^. In our simulations, the excitation of LSPRs also lead to increased transmission with respect to pristine Au film due to increased evanescent coupling of the incident light to the back surface.

**Figure 4:**
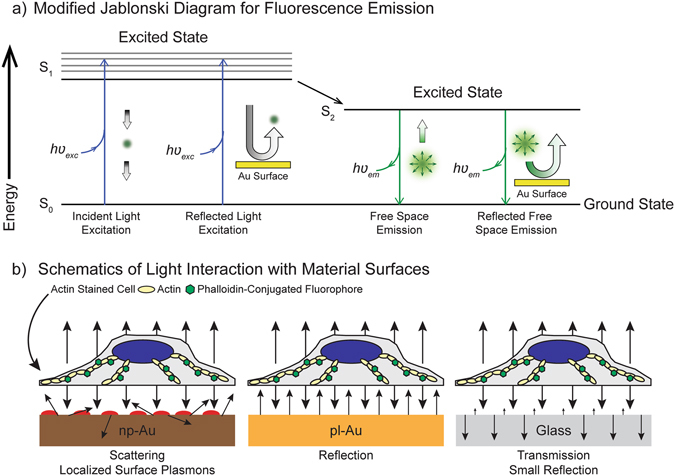
(**a**) The effect of the surfaces on fluorescence light emission efficiencies is illustrated through modified Jablonski diagram. Although the efficiencies of different processes depend on material properties, the general scheme could be summarized as follows. Fluorophores are excited to the excited state (S_1_) through both incident and reflected light from the substrate. Excited electrons relax to a lower energy state (S_2_) after solution relaxation processes (indicated by solid arrow). From this lower energy state (S_2_) the fluorescence emission occurs as the electrons return back to ground state (S_0_). The light emitted from fluorophores is collected directly (fluorophores are point light sources) and after reflecting from the substrate. (**b**) The decrease in observed intensity between np-Au and pl-Au is associated to the nanostructured surface of np-Au causing differences in the interactions of the emitted light and the material surface (scattering and localized surface plasmons).

More specifically, the problem is formulated as follows. Depending on the reflectivity and scattering characteristics of the substrate, both the excitation and collection efficiencies could be enhanced. This is illustrated in a modified Jablonski diagram (Fig. 4a)^27^. For low intensities used in these experiments, only a small fraction of fluorescence molecules are excited to the higher energy state (S_1_). Accordingly, any enhancements in the incident light intensities will lead to linearly increased excitation efficiencies to the higher energy state (S_1_). When fluorophores are residing on a reflective surface, both the incident and reflected light excites electrons to the higher energy state (Fig. 4a). This is particularly noticeable for Alexa Flour 555 since the light reflection from pl-Au is nearly 400% higher than the absorption on average within the excitation wavelength range of Alexa Flour 555 (Fig. 3b). The excited electrons relax to a lower energy state (S_2_) through solution relaxation processes. Subsequently, as the electrons residing on lower excited energy state (S_2_) return the ground state (S_0_), fluorescent light is emitted. A portion of this emission directly travels back to the microscope objective in epifluorescence mode, while some portion travels down to the material surface, where the light is either reflected, absorbed, or transmitted (Fig. 4b). Since the pl-Au substrate is highly reflective; a larger collection efficiency is expected for fluorophores residing on pl-Au substrates. Accordingly, increased fluorescence emission on pl-Au substrates is a result of both excitation (S_0 _→ S_1_) and collection (S_2_ → S_0_) efficiency enhancements due to the highly reflective characteristics of this material. One can incorporate these far-field effects on fluorescence signal using reflectance characteristics of the substrate as in:

1 $$E{F}_{Fluor}={\iiint }_{{\lambda }_{exc,}{\lambda }_{em},\theta }(1+R({\lambda }_{ext},\theta ))(1+R({\lambda }_{em},\theta ))$$

Here, the reflection coefficient *R* depends on both wavelength and incidence angle on the substrate. The fluorescence enhancement factor *EF*_*Fluor*_ is due to the far-field effects as indicated in modified Jablonski diagram in Fig. 4a and should not be confused with metal enhanced fluorescence (MEF) related to the excitation of surface plasmons. MEF is a near-field interaction effect, where the plasmonic particles boost the excitation and emission efficiencies through near-field coupling effects^28^. On the pl-Au substrate considered here, the low incidence angles of excitation light prevent excitation of surface plasmons. Hence, pl-Au surface signal enhancement is due to purely far-field effects and could be related to reflection and absorption characteristics of the Au substrate. On the other hand, the lower fluorescence signals obtained on glass substrates are attributed to lower reflection (and higher transmission) coefficients of the glass substrates (Fig. 3a). A surprising result is the even lower fluorescence signals obtained from np-Au substrates relative to the glass substrate. This could be associated to weaker but existing fluorescence enhancement effects on glass substrates. As shown in Fig. 3c, a small fraction of incident light is reflected back from the glass surface for varying angles of incidence. In addition to excitation field enhancements due to these reflections, an objective with a numerical aperture (NA) equal to 0.5 (i.e., the objective used for these fluorescence measurements) will collect a portion of the nearly uniformly radiated light falling within the collection cone half-angle (θ) of 30 degrees. Hence, it is expected that a larger portion of the emitted light will be collected due to reflections at water/glass interface. This leads to an increased fluorescence signal on glass substrates relative to free space emission. On np-Au surfaces, fluorescence behavior is closest to free space emissions, since the light reflection at water/np-Au surface is minimal due to the randomized strong light scattering through LSPRs^10, 11^ and strong losses in np-Au material as shown in Fig. 3b and d.

**Figure 5:**
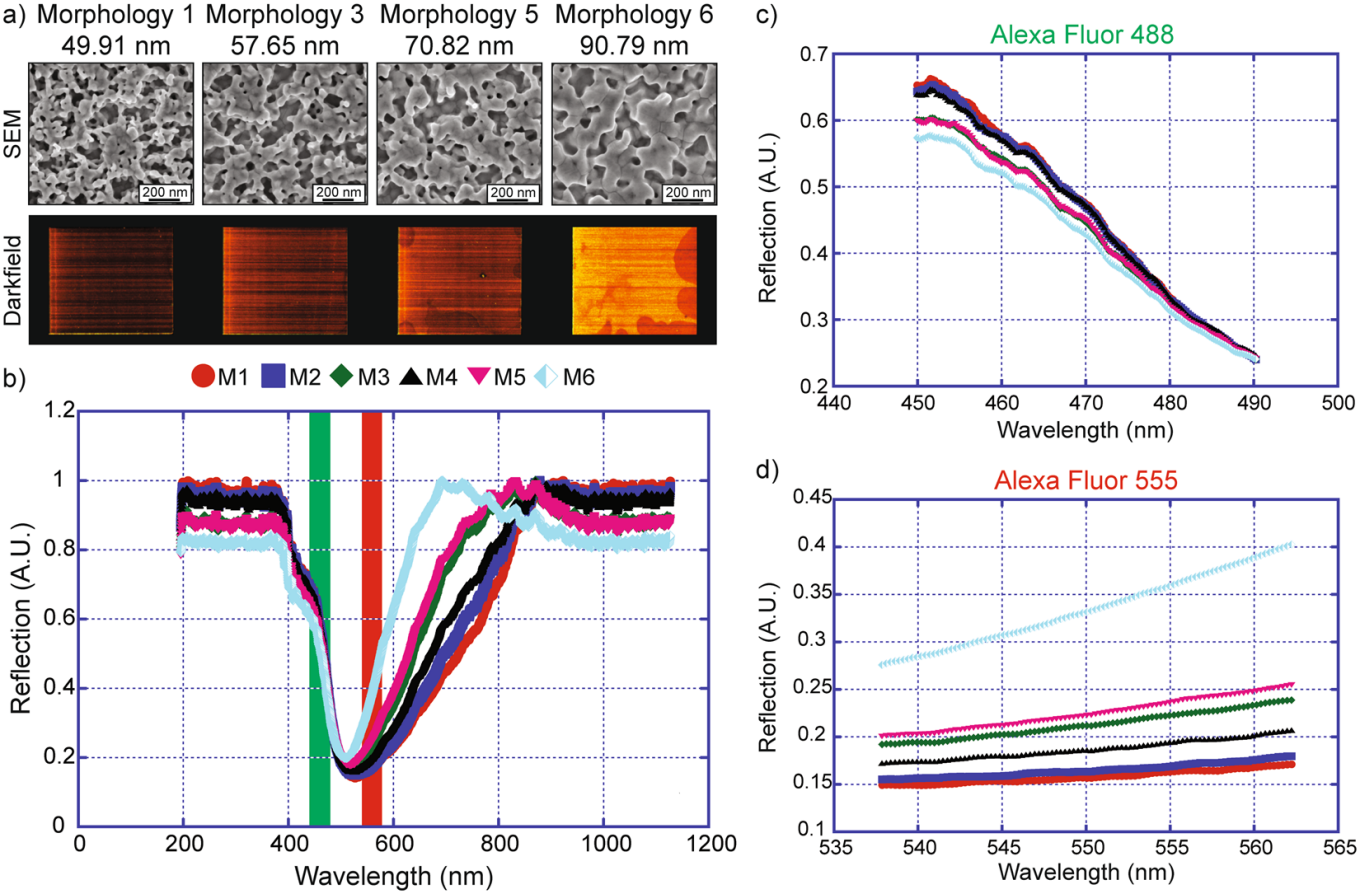
(**a**) High magnification scanning electron micrographs show the nanostructure of np-Au films with varying morphologies representing a wide range of feature sizes. The corresponding dark field images show an increase in the perceived brightness of gold as feature size is increased. (**b**) A large overall shift towards higher reflection between wavelengths of approximately 500–800 nm is seen as feature size is increased. (**c**) Only small changes in reflection are observed in the Alexa Fluor 488 wavelength range. (**d**) Large increases in reflection are seen in the Alexa Fluor 555 wavelength range.

We verified this claim using reflection dark field spectroscopy measurements (Figure S1) and quantified the intensity of scattered light from the np-Au surface over a broad wavelength range. A microfabricated library of varying np-Au morphologies^29^ was used to study the effect of feature sizes on scattered light spectra (Fig. 5a). Here the dark field signal is normalized to unity and represented in arbitrary units (A.U.). A significant drop in dark field scattered signal is observed within the wavelength range of ~500 to 800 nm (Fig. 5b). This drop in reflectivity is consistent with excitation of LSPRs as recorded previously on np-Au samples^18–21^ and in agreement with our FDTD simulations (Fig. 3b). Even for np-Au with a small ligament size (~49 nm – M1), a strong drop in dark field signal is observed for both excitation and emission wavelengths of the fluorophores considered here (Fig. 4c and d). These observations are attributed to losses inside the np-Au material due both the presence of LSPR (due to nanostructure) and the material properties of gold. In other words, these experiments show that due to the presence of nanostructure, np-Au substrates behave much like an open boundary (sink) similar to free space fluorescence emission. As a result, weaker fluorescence signals on np-Au substrates with respect to glass substrates are observed.

**Figure 6:**
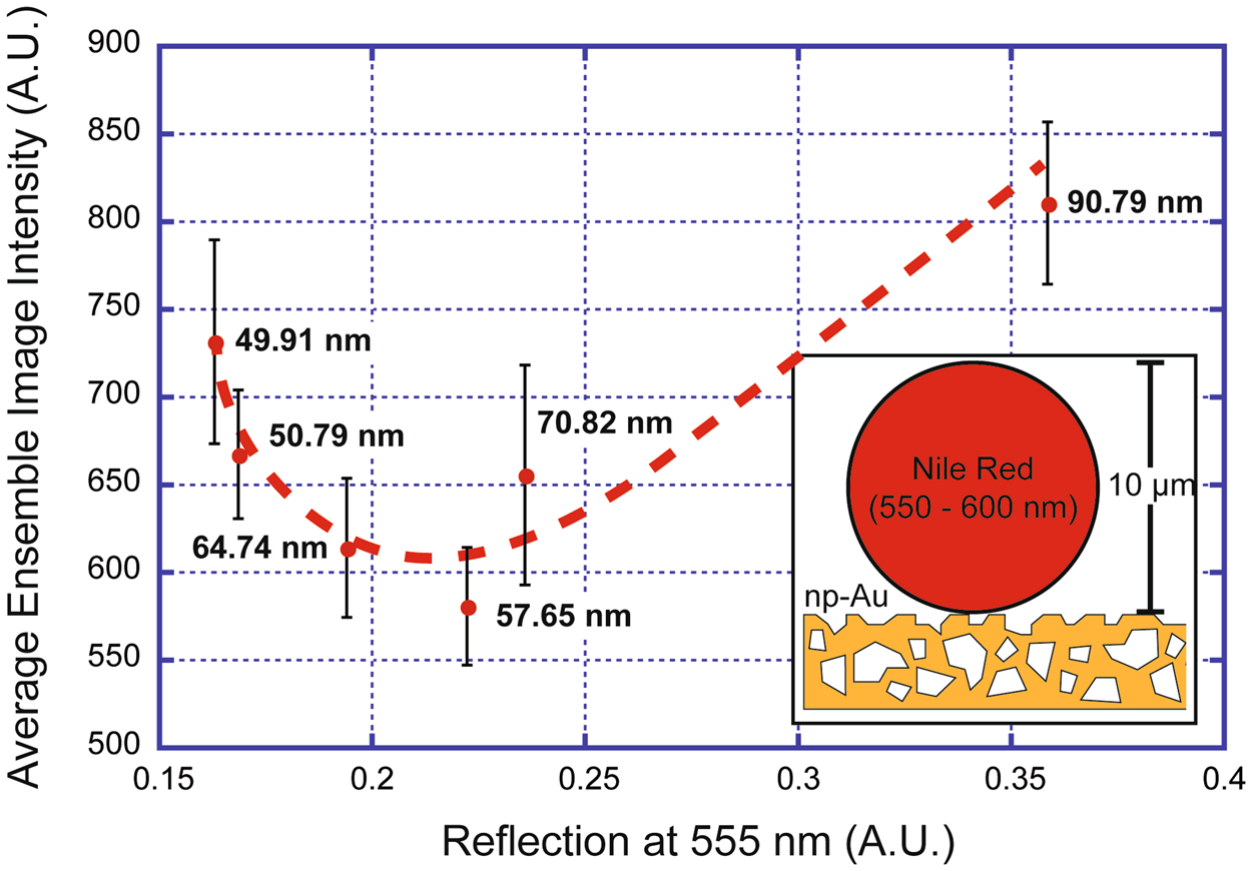
Correlation between np-Au reflection at 555 nm and the observed fluorescence intensity: Fluorescent Nile Red microspheres with a diameter of 10 µm were dispersed on the surface of a np-Au feature size library (inset). The intensity from each morphology previously measured by reflection dark field spectroscopy was observed at a constant exposure time of 50 ms. The mean ensemble-average of the observed fluorescence intensity is plotted as a function of the measured reflection at 555 nm (error bars represent standard deviation). The previously measured feature size for each point is indicated at each measurement point. While dark field reflection had a linear dependence on the feature size, fluorescence intensity exhibited a non-monotonous trend with respect to feature size with an inflexion around 50 nm to 65 nm. (Red dashed line is only for visual aid and not a fit).

In order to provide further insight into the influence of nanostructure on fluorescence intensity for the red spectrum, we used fluorescent (Nile Red) microspheres of approximately 10 µm in diameter and excitation wavelength of 550 to 600 nm (Fig. 6 inset). By uniformly dispersing the fluorescent beads on the np-Au morphology library that displays feature sizes ranging from 50 nm to 100 nm^29^, we determined the average fluorescence intensity of a group of spheres located on each different np-Au morphology via epifluorescence microscopy and image analysis, as done previously. These values are plotted with respect to the previously measured reflection magnitude (at 555 nm), both as a function of ligament size (Fig. 6). Overall, the coarser np-Au morphologies (similar to patches of pl-Au) lead to significantly more fluorescence intensity for the spheres. However, the fluorescence intensity displayed a non-monotonous dependence on feature size with an inflexion at between 55 nm to 60 nm. Outside of this feature size range (Morphology 1 and 2) a linear dependence that matched the measured reflection was observed.

Although there is a non-linearity in the observed fluorescence intensity as a function of feature size, the overall change in intensity from standard np-Au feature size (~30 nm) to higher red fluorescence with increasing feature size is consistent with the measured changes in material reflection. The non-linearity observed in the intensity at a feature size around 50 nm suggests that, for the case of the polystyrene bead, the interaction of the fluorophores with the nanostructure is more complex than just a scattering or reflection mechanism.

## Discussion

In this work, we have shown that fluorescence intensity from cell-conjugated fluorophores is significantly altered due to the far-field interactions of the emitted light with nanostructured materials. Enhancements in fluorescence intensity caused by the presence of strong LSPRs have been previously reported on np-Au films^30^. However, previous studies have primarily focused on surface functionalized nanometer sized fluorescent species such as quantum dots or unconjugated fluorescent dyes^30–33^. In contrast, we have demonstrated a wavelength- and feature size-dependent specific reduction in fluorescence on np-Au surfaces from fluorophores functionalized hundreds to thousands of nanometers away from the surface. LSPRs have been implicated as the driving factor in metal fluorescence enhancement (MEF) due to the strong fields created in the nanostructures^10, 34^. The ideal distance for seeing strong effects from MEF is at a distance of 5–8 nm from the surface of the plasmonic structure^10, 35^. After this distance the enhancement of fluorescence exponentially diminishes and is no longer observed over 50 nm away from the plasmonic surface. Therefore, although LSPRs are with no doubt present in the np-Au samples tested here (seen in the reflection shift of Fig. 5b), the distance of the cell-bound fluorophores suggests that neither excitation nor extinction is effected by near-field enhancement due to the LSPRs for fluorophores located far from the substrate. However, the non-linear dependence of observed fluorescence on nanostructure illustrated in Fig. 6 does suggest that some signal enhancement due to MEF may be contributing to the measured signal. It is possible that although the polystyrene bead is larger than a cell, fluorescence in the area of the bead that sits directly on the surface of the np-Au gets significantly enhanced due to LSPRs. This would be consistent with previous findings showing significant MEF in the regime of 10–30 nm pore size, but a sharp reduction as pore size is increased further from dropping plasmonic coupling quality factor (diminishing field enhancements in larger feature voids)^36^. Above approximately 50 nm the MEF is reduced to a point that fluorescence is primarily dependent on the scattering as predicted by the shift in reflection spectra^10^.

Through this study we have demonstrated, in a cell type-independent manner, that relative protein quantification across differing material nanostructures via fluorescent markers must consider the effects of the underlying substrate. Additionally, utilizing large fluorescent polystyrene beads as a simple model of cell coverage on the np-Au surfaces (Fig. 6), we have decoupled the influence of cell type while observing similar results as in the cell-based studies (Figs 2 and 3). Similar results were seen using two different dye types (Alexa Fluor 555 and Nile Red) which strongly suggests that the observed effect is purely due to the np-Au nanostructure acting like an open boundary similar to free-space fluorescence emission (Fig. 4). We show that the observed enhancement effects are not due to the unique nature of the fluorescence molecules used here. Instead, they are related to far-field effects created by the spectrally varying reflectance at material boundaries (water/substrate). An important implication of this is that the effect acts independently of the fluorophore and depends primarily on: a) the scattering relative scattering/reflection characteristics of the substrate, and b) the proximity of the fluorophore to the nanostructured surface.

Consequently, utilizing epifluorescence for quantifying differential cellular behavior between separate nanostructured materials will likely lead to significant errors. In order to prevent such artifacts, guided by the results presented here, we stress the importance of characterizing the optical properties of each material surface before measurement. Once a material surface has been quantified a fluorophore less sensitive to the range of feature sizes on the material surface can be used for labeling. However, as seen in Fig. 6, regardless of scattering effects the material surface can lead to altered fluorescence depending on the proximity of the fluorophore from the LSPR region (within 50 nm from the surface). Therefore, on nanostructured materials, both far-field and near-field effects should be considered. Ultimately, this makes the cross-comparison between nanostructured materials exceedingly difficult, when angular and wavelength dependence is also considered as in Eq. 1. The best strategy to account for this difficulty is to reserve fluorescence quantification to comparisons between two identically nanostructured samples, and use more rigorous biochemical assays such as western blotting for cross comparison between differently nanostructured materials.

## Methods

### Sample Fabrication

The np-Au samples were produced by successively sputtering-depositing 160 nm-thick chrome, 80 nm-thick gold, and 600 nm-thick gold-silver (64% silver and 36% gold, atomic %) on a 0.15 mm-thick glass cover slip (5 mm diameter spots onto a 12 mm diameter coverslip) through a laser-cut elastomer stencil. The final np-Au was obtained by selectively dissolving silver in heated nitric acid, while gold atoms self-assemble into the three-dimensional ligament network. The pl-Au samples were prepared similarly by depositing 160 nm-thick chrome and 200 nm-thick gold. The library of varying np-Au morphologies was fabricated by first photolithographically patterning the np-Au precursor on a silicon chip and subsequently dealloying it to produce the np-Au patterns. These patterns were than annealed by exposure to a focused laser beam (5 µm spot size) rastered at 500 μm s^−1^ with laser irradiance varying between 5.09 MW/cm^2^ and 3.50 MW cm^−2^ in approximately 0.32 MW cm^−2^ increments.

### Cell Culture and Staining

NIH/3T3 (embryonic mouse fibroblasts – ATCC CRL-1658) were purchased from American Type Culture Collection (Manassas, VA). Fibroblasts were grown on tissue culture plastic using DMEM (high glucose with sodium pyruvate – catalog number 11995073, ThermoFisher, Carlsbad, CA) supplemented with 10% fetal bovine serum (ThermoFisher) and 1% penicillin/streptomycin (ThermoFisher). Once approximately 70% confluent, cells were stained with trypan blue (ThermoFisher) and viable cells counted using a hemocytometer (Sigma-Aldrich, St. Louis, MO) then plated at equal densities (~100 cells/mm^2^) on the test surface materials. Fibroblasts on all surface materials were grown until approximately 70% confluent before being either stained for imaging or lysed for western blotting.

To stain cells for f-actin, cultures were first fixed with warm 4% paraformaldehyde (Affymetrix, Santa Clara, CA) for 20 minutes. Fixed cells were then rinsed twice with PBS[+] (containing Mg^2+^ and Ca^2+^) before being washed with 0.1% Triton X-100 in PBS[+] (Sigma) for 5 minutes. After exposure to Triton X-100, cells were washed again with PBS[+] and blocked using 10% fetal bovine serum in PBS[+] for 30 minutes at room temperature. After blocking, cells were incubated with 1:50 concentration dilution of phalloidin (Molecular Probes/ThermoFisher) staining solution in PBS[+] for 30 minutes at room temperature in the dark. Cells were then washed three times with PBS[+] counterstained with 0.25 µg/mL DAPI (ThermoFisher) for 5 minutes and mounted using ProLong Antifade Gold (ThermoFisher) onto No. 1.5 slides (Ted Pella, Redding, CA). All images were taken using a Zeiss Observer D1 inverted fluorescence microscope.

### Western Blot and Analysis

Fibroblasts were lysed and collected in cold lysis buffer (150 mM sodium chloride, 1.0% NP-40, 0.5% sodium deoxycholate, 0.1% SDS 50 mM Tris pH 8.0) containing Halt protease inhibitor (ThermoFisher). Samples were sonicated for 5 minutes in a chilled water bath and centrifuged at 14,000 × g at 4 °C for 15 minutes. Supernatant was collected and protein concentrations were quantified using the Pierce BCA Assay (ThermoFisher) according to the manufacturer’s protocol. Proteins were separated on a 12% Bis-Tris Plus gel (ThermoFisher) and transferred to a PDVF membrane using the iBlot dry blotting system (ThermoFisher). Membranes were blocked for 1 hour with Odyssey buffer (LI-COR) and reacted overnight at 4 °C with antibodies specific for β-actin (Sigma, St. Louis, MO) and GAPDH (Cell Signaling, Danvers, MA) both diluted 1:1000 in blocking buffer. After washing in PBS to remove the primary Abs, membranes were incubated with secondary antibodies conjugated to infrared dyes 700 and 800 (Rockland) for 1 hour at room temperature. Immunoreactive blots were visualized and densitometry was performed using the Odyssey Infrared Imaging system (LI-COR).

### Reflection Dark Field Spectroscopy

Optical scattering properties of nanoporous gold (np-Au) thin films were studied with an optical reflection dark-field microscope (Nikon) equipped with a high-sensitivity spectrometer (Ocean Optics HR4000), as illustrated in Figure S1. The np-Au morphology library was placed face-up onto the sample holder of the microscope. The source light is directed to the np-Au surface along the side of the 100x objective. Part of the excitation light is backscattered at the np-Au surface and to the center by a ring-shaped mirror near the end of the objective; the other part is absorbed in or transmitting through the np-Au film. The schematic shows how the backscattered light spectrum of each finite-sized np-Au thin film was measured. All the obtained scattering light spectra were normalized to the excitation light spectrum, and the boxcar pixel smoothing algorithm was used.

## Electronic supplementary material

Supplementary Info
