# Supplementary material for: Nanostructure Introduces Artifacts in Quantitative Immunofluorescence by Influencing Fluorophore Intensity
Source: Sci Rep. 2017 Mar 27;7:427. doi: 10.1038/s41598-017-00447-7 (PMC5428417; doi:10.1038/s41598-017-00447-7)
Supplement: Supplementary file 1 — Supplementary Info [file 41598_2017_447_MOESM1_ESM.pdf]

**Supporting Information for:**

# Nanostructure Introduces Artifacts in Quantitative Immunofluorescence by Influencing Fluorophore Intensity

Christopher A. R. Chapman[1], Xiangchao Zhu[2], Hao Chen[3], Ahmet A. Yanik[2], Pamela J. Lein[3], & Erkin Seker[4*]

[1]Department of Biomedical Engineering, University of California – Davis, Davis CA 95616

[2]Department of Electrical Engineering, University of California – Santa Cruz, Santa Cruz, CA 95064

[3]Department of Molecular Biosciences, University of California – Davis, Davis CA 95616

[4]Department of Electrical & Computer Engineering, University of California – Davis, Davis CA 95616

## Reflection Dark Field Spectroscopy Setup

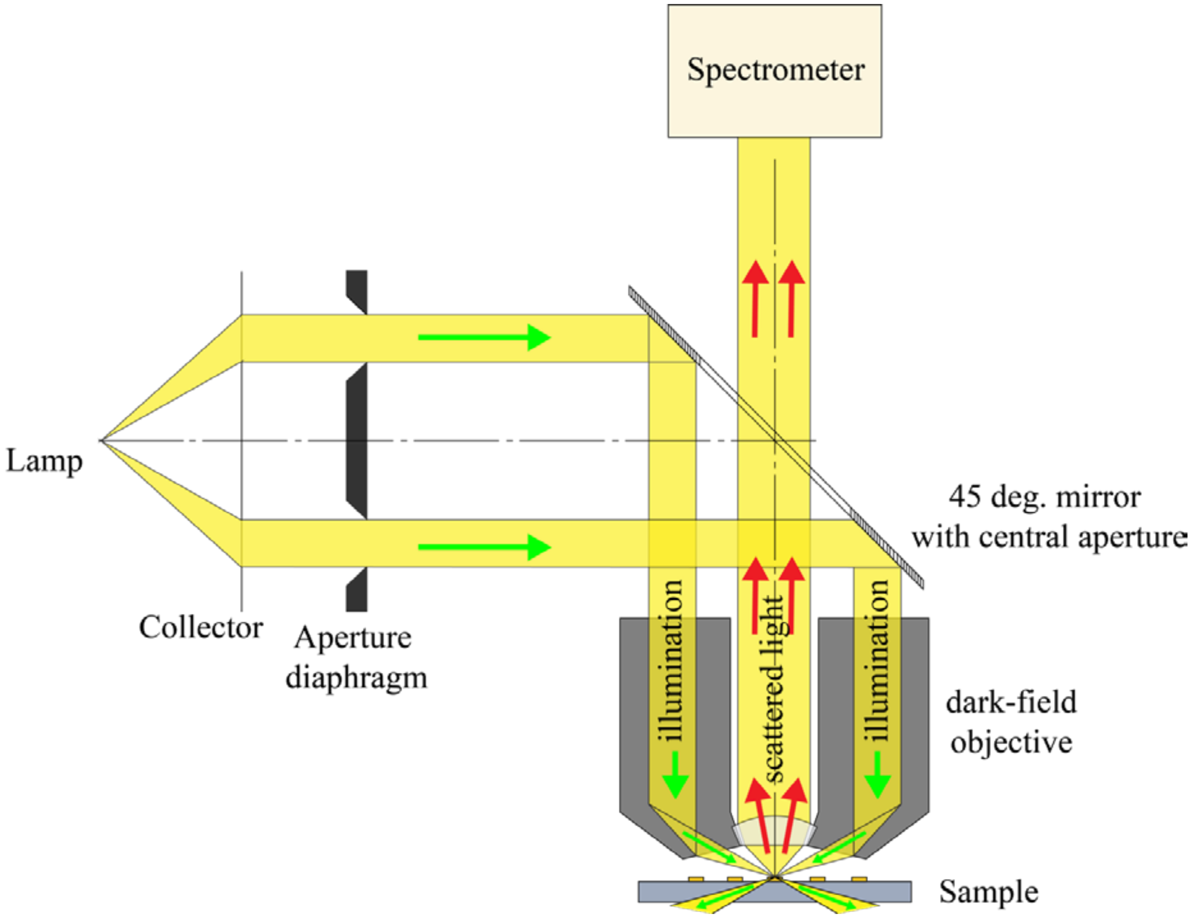


**Figure S1** – Reflection dark field spectroscopy setup. The specimen is illuminated by white light source. The illumination light is backscattered from the np-Au surface and directed through the objective to the spectrometer. The dark field scattering spectra of different np-Au samples were measured by the spectrometer.

## Full-Length Western Blots

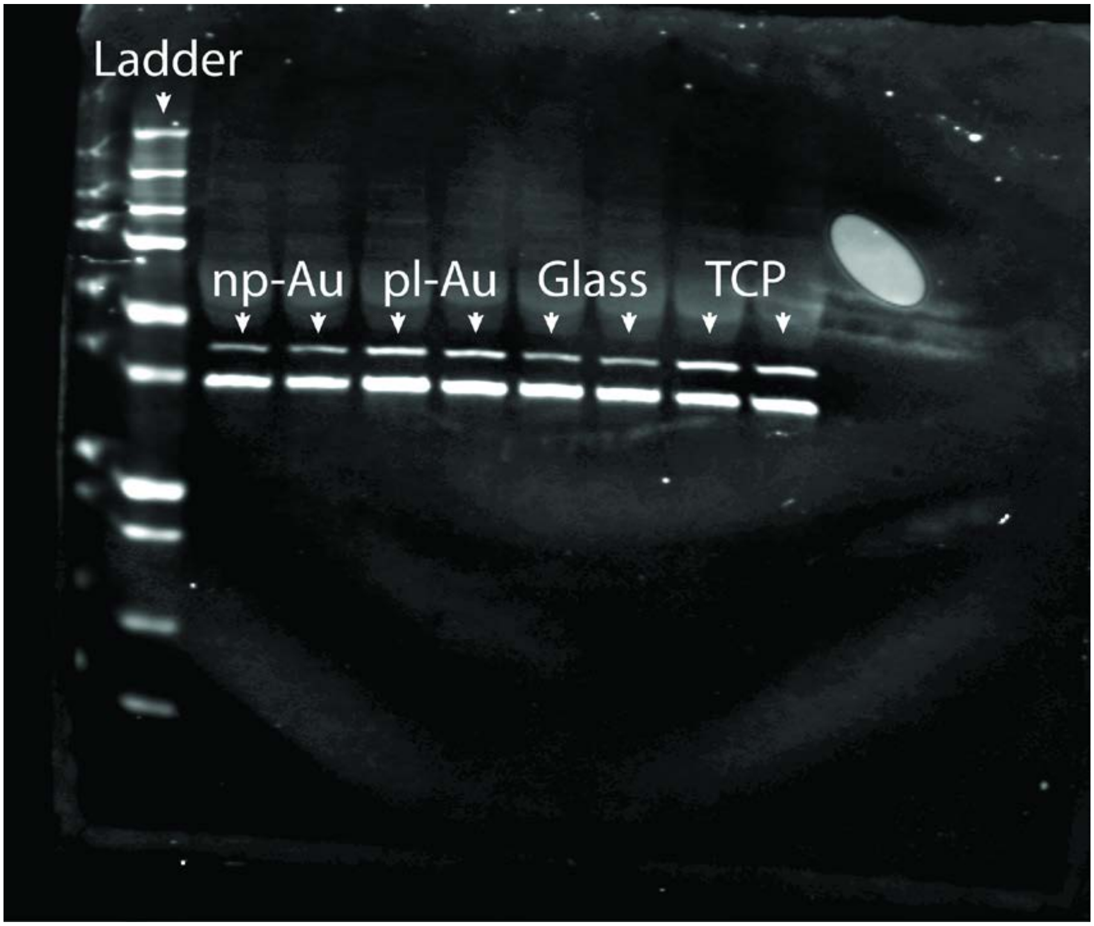


**Figure S2** – Image of the unprocessed western blot gel with lane 1 consisting of the standard ladder, lanes 2 and 3 consisting of cell lysate from nanoporous gold (np-Au) samples, lanes 4 and 5 consisting of cell lysate from planar gold (pl-Au) samples, lanes 6 and 7 consisting of cell lysate from glass samples, and finally lanes 8 and 9 consisting of cell lysate from tissue culture plastic (TCP). The gel was stained using antibodies against ß-actin (top band) and GAPDH (bottom band). The gels were then visualized using appropriate secondary antibodies. Please see the methods section for more specific details on the antibodies and systems used for performing the western blot.

**Image Analysis in MATLAB**

```
clear all
clc

filename = sprintf ('%s','…'); %change the name of the file here

A = imread(filename);

imsize = size(A);

count = 0;
tot_int = 0;

black = min(min(A));
for i = 1:imsize(1)
    for j = 1:imsize(2)
        if A(i,j) <= black

        end

        if A(i,j) > black
            count = count + 1;
            k(count) = A(i,j);
        end
    end
end

ens_int = sum(k)/count;
```
